# INTERGENERATIONAL PERCEPTIONS OF FAMILY DYNAMICS AMONG OLDER PARENTS AND ADULT CHILDREN ON THE FACES IV AND SFI II

**DOI:** 10.1093/geroni/igac059.2331

**Published:** 2022-12-20

**Authors:** Jessica Hahne, Eleanor A Skemp, Brian Carpenter

**Affiliations:** Washington University in St. Louis, St. Louis, Missouri, United States; Washington University in St. Louis, St. Louis, Missouri, United States; Washington University in St. Louis, St. Louis, Missouri, United States

## Abstract

Standardized measures of family dynamics are rarely used with late-life families in either research or clinical contexts. The purpose of this study was to examine perceptions of family dynamics from the perspective of older parents and their adult children on two widely used family assessment measures. To our knowledge, no previous studies have reported data on these measures with late-life families. Within a larger project examining decision-making in late life, 34 older parents and two of their adult children (total N= 102) completed the Family Adaptability and Cohesion Evaluation Scale (FACES) IV and the Self-Report Family Inventory (SFI) II. Parents were mostly mothers (94.1%; M age= 75), but adult children included both daughters (67.6%; M age= 46) and sons (32.4%; M age= 46). For comparison with published norms, we report subscale scores on both family measures for parents and adult children. The mean intrafamily ICC (two-way, mixed effects, single rater, absolute agreement) for individual items for the FACES IV was .561 (SD= .164, Min= .128, Max= .827), and the mean intrafamily ICC on the SFI II was .422 (SD= .178, Min= .014, Max= .750). Overall, family consistency was in the poor to moderate range, but with a high degree of variability across families. Moreover, within families, parents and children had highly variable inconsistency in their perception of family dynamics. Findings from this study provide insight as to how established measures function in late-life families, and they highlight the importance of assessing multiple family members in order to understand family dynamics.

